# Ultrasound in active surveillance for low-risk papillary thyroid cancer: imaging considerations in case selection and disease surveillance

**DOI:** 10.1186/s13244-021-01072-9

**Published:** 2021-09-16

**Authors:** Sangeet Ghai, Ciara O’Brien, David P. Goldstein, Anna M. Sawka, the Canadian Thyroid Cancer Active Surveillance Study Group, Lorne Rotstein, Dale Brown, John de Almeida, Patrick Gullane, Ralph Gilbert, Douglas Chepeha, Jonathan Irish, Jesse Pasternak, Shereen Ezzat, James P. Brierley, Richard W. Tsang, Eric Monteiro, Afshan Zahedi, Jacqueline James, Ian Witterick, Karen Gomez Hernandez, Antoine Eskander, Danny Enepekides, Kevin Higgins, Ilana J. Halperin, Afshan Zahedi, Karen Devon, Everton Gooden, Manish Shah, Mark Korman, Janet Chung, Kareem Nazarali, Eric Arruda, Artur Gevorgyan, Michael Chang, Sumeet Anand, Vinay Fernandes, Denny Lin, Avik Banerjee, Vinita Bindlish, Vinod Bharadwaj, Maky Hafidh, Raewyn Seaburg, Laura Whiteacre

**Affiliations:** 1 Joint Department of Medical Imaging, University Health Network – Mount Sinai Hospital – Women’s College Hospital, University of Toronto, Toronto, ON Canada; 2 1PMB-283, Toronto General Hospital, 585 University Avenue, Toronto, ON M5G 2N2 Canada; 3 Princess Margaret Cancer Centre, Department of Otolaryngology-Head and Neck Surgery/Surgical Oncology, University Health Network and University of Toronto, Toronto, ON Canada; 4 Division of Endocrinology, Department of Medicine, University Health Network and University of Toronto, Toronto, ON Canada

**Keywords:** Watchful waiting, Papillary cancer, Thyroid gland, Ultrasonography, Management

## Abstract

Active surveillance (AS) of small, low-risk papillary thyroid cancers (PTCs) is increasingly studied in prospective observational studies. Ultrasound is the primary imaging modality for case selection. While researchers have put forward selection criteria for PTCs based on size, absence of suspicious lymph nodes and tumor location, there are limited reported data highlighting inherent ultrasound limitations and guidelines for case selection and follow-up. We report our experience including imaging limitations encountered in the ongoing AS prospective observational study for PTCs measuring < 2 cm at our institute. We define disease progression as an increase in size of > 3 mm in the largest dimension of nodule or evidence of metastatic disease or extrathyroidal extension. Accurate, consistent and reproducible measurements of PTCs are essential in risk stratifying patients for the option of AS or disease progression. Interobserver discrepancy, shadowing from coarse calcification and background parenchyma heterogeneity or thyroiditis can limit accurate PTC size assessment and therefore hinder patient eligibility evaluation or AS follow-up. Following the ACR Thyroid Imaging, Reporting and Data System (TI-RADS) protocol of three-axes technique to measure a thyroid nodule enables reproducibility of measurements. In patients with multi-nodular goiter, accurate identification and labeling of the PTC is important to avoid mistaking with adjacent benign nodules at follow-up. Ultrasound assessment for extrathyroid extension of PTC, and relationship of PTC to trachea and the anatomic course of the recurrent laryngeal nerve are important considerations in evaluation for AS eligibility.

## Key Points

- Active surveillance (AS) of small, low-risk PTCs is a strategy that may mitigate potential overtreatment of low-risk PTC.
- Meticulous ultrasound evaluation is integral in terms of patient selection as well as follow-up of patients on AS.
- Accurate, consistent and reproducible PTC measurements are essential.
- Heterogenous background parenchyma from thyroiditis and coarse calcification with acoustic shadowing in PTC can limit accurate nodule assessment.
- All nodules should be assessed carefully for nodal disease, extracapsular extension, relationship to the trachea and the anatomic course of the recurrent laryngeal to evaluate eligibility for AS.

## Background

The incidence of thyroid cancer has rapidly increased in the past few decades. Thyroid cancer is the 8th most common malignancy worldwide with a very low mortality risk [1]. The increased incidence of thyroid cancer is primarily explained by small papillary thyroid cancers (PTCs) which has been attributed to the worldwide increased use of ultrasonography. The majority of these PTCs are low-risk tumors of an indolent nature [2]. Autopsy series have reported a prevalence rate of 4–11% of PTC in the general population, signifying that a significant number of patients died with but not from thyroid cancer [3, 4]. Given the high prevalence rate and very low mortality risk of small PTC confined to the thyroid, there is a large disease reservoir that has been uncovered due to ultrasound imaging. As a result, active surveillance (AS) of small, low-risk PTCs has become a topic of multiple prospective trials around the world in order to mitigate potential overdiagnosis and overtreatment of low-risk PTC [5–9]. Ultrasound is established as the best modality for detecting and characterizing thyroid nodules and therefore is the primary modality used for case selection in AS enrollment. Recently published consensus statement from the Japan association of endocrine surgery task force on management for papillary thyroid microcarcinoma highlights the need of multidisciplinary team approach to accurately evaluate PTCs at beginning and during AS [10]. On May 11, 2016, we initiated a prospective observational study (Clinicaltrials.gov: NCT03271892) at our institute offering AS or surgery to patients with low-risk PTC [1]. This study is approved by the University Health Network Research Ethics Board (#15-8942) and participants consented to study participation. In this ongoing study, we include patients 18 years of age or older, with thyroid nodules that are PTC or suspicious for PTC on fine needle biopsy and < 2 cm in maximum diameter in the absence of nodal metastasis, extrathyroidal extension, other significant indications for thyroid or parathyroid surgery or tumors based on location that would be considered at high risk of invasion into the trachea or recurrent laryngeal nerves (RLN). As of October 30, 2020, one hundred and eighty-two patients had been enrolled in the study [140 females, 42 males; mean age 52 years] [11].

Meticulous ultrasound evaluation is integral in terms of patient selection as well as follow-up. In most thyroid PTC AS trials, indications for surgery are based on US features including identification of nodal metastases as well as growth of the tumor. Sasaki and colleagues from Kuma Hospital, Japan, recently reported that conversion surgery in their AS cohort was significantly less in the second half of study over a 15-year period and attributed this to physician’s confidence and patients’ trust and understanding of the disease [12]. Standardization of ultrasound technique and interpretation with understanding of its limitations in a multidisciplinary approach at the very onset of AS program has the potential to further enhance patient and physician confidence in AS for small PTCs. In this paper, we report our experience and the imaging limitations we encountered in the ongoing AS prospective observational cohort study at our institute.

### Thyroid ultrasound

All patients being considered for enrollment in the prospective cohort study underwent ultrasound imaging the Joint Department of Medical Imaging (University Health Network, Sinai Health System and Women’s College Hospital) at the University of Toronto. The baseline thyroid scans evaluated the entire thyroid gland with focus on the primary PTC and cervical lymph nodes for inclusion. As per department guidelines, up to 4 nodules with the highest risk/suspicion category are documented at the time of the scan. Apart from recording size of the PTC in 3 dimensions and if the nodule was along the course of recurrent laryngeal nerve (RLN) or immediately adjacent to the trachea, the thyroid ultrasound also examined for extrathyroidal extension (ETE) of the PTC or evidence of nodal metastases. Studies have shown ultrasound to be beneficial in diagnosis of ETE [13, 14], whether to strap muscles, trachea or RLN, which determines if AS is appropriate or not. Following inclusion in the study, all patients have 6-month interval scans through the department for the first 2 years and then yearly after to document change in size, relationship with sensitive structures (RLN or trachea) or nodal disease.

### Nodule location

**Fig. 1:**
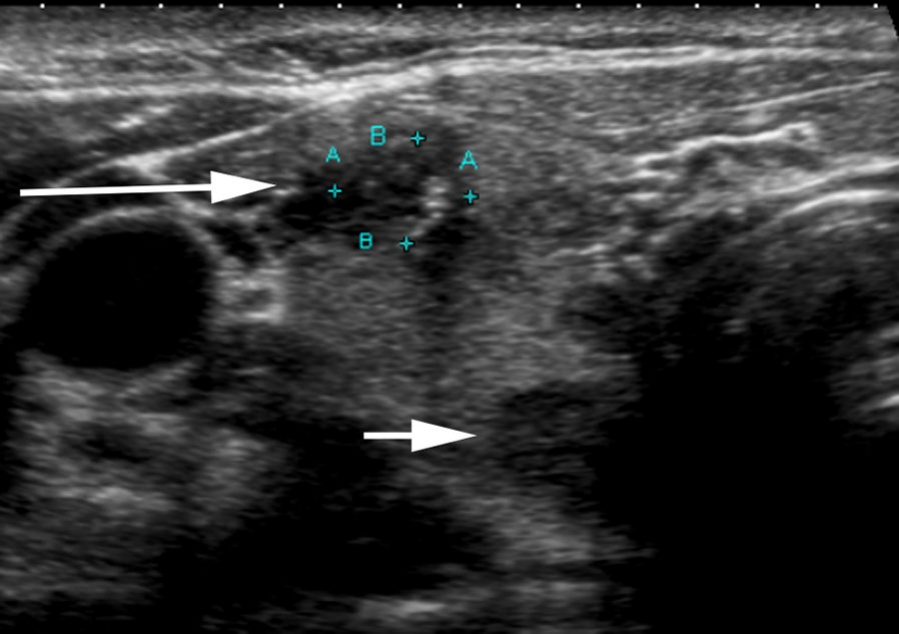
52-Year-old female with multi-nodular goiter and 6 mm PTC in upper pole of right lobe of gland (long arrow). The PTC nodule is hypoechoic with irregular margins and microcalcification. An adjacent similar sized hypoechoic benign nodule with well-defined margins (short arrow) is seen in the posterior aspect of upper pole of right lobe of thyroid gland. It is important to meticulously document the location of the PTC (in this instance as right, upper, anterior) to prevent mistaking an adjacent benign nodule for the PTC at follow-up

It is important to identify the location of PTC accurately for comparison at follow-up. In patients with multi-nodular goiter (MNG), more than one nodule may be present in a segment of the gland harboring the PTC. In large teaching clinical departments wherein follow-up scans are likely to be performed by different operators at each follow-up visit, there is potential of mistaking one of the adjacent benign nodules in the same segment of the gland as PTC, if not accurately labeled or identified at baseline (Fig. 1). The location of the PTC should be meticulously documented in the report (right, left, isthmus, upper, mid, lower and if required lateral, medial, anterior or posterior). Saving video clips in transverse and sagittal planes for both lobes can provide valuable spatial relationship between the PTC and other adjacent benign nodules. While this is done routinely at our academic center, we recognize that it may not be feasible in other practices because of storage and memory limitations with picture archiving and communications systems (PACS). However, saving video clips at least for the complex cases (such as in the case of patients with multiple nodules) enables accurate identification and localization of the PTC in relation to other adjacent nodules. In our experience, assigning a TIRADS score to the PTC at baseline also helps in correctly identifying the known PTC at follow-up, since the adjacent < 2 cm benign nodules in MNG are likely to have lower point scores compared to the PTC which tend to have ACR-TIRADS score of 4 or 5 to have triggered a biopsy. Additionally, saving key images of the PTC as well as of the adjacent nodule/s on PACS with the image number displaying the PTC on the report will enable distinguishing between the PTC under AS and other adjacent nodules at follow-up.

### Nodule size

The AS protocol in our study allows inclusion of nodules < 2 cm in maximum dimension and defines an increase in size of the PTC by > 3 mm in largest dimension as disease progression prompting a surgical recommendation (where tumor growth is confirmed by independent review by a study radiologist (S.G.) on two consecutive ultrasound examinations, generally performed within a few months of each other). The rationale for confirming tumor growth on two consecutive measurements is to mitigate the risk of measurement error, particularly in cases where there are no other indications for surgery (i.e. in absence of incident nodal disease or extrathyroidal extension). Of note, Ito et al. recently reported that in papillary microcarcinomas where the diameter was reported to increase ≥ 3 mm, a subsequent reduction in size was observed in 37 cases (47.4%) with continued observation. The authors thus suggested that immediate surgery following nodule enlargement of ≥ 3 mm may be premature for patients with papillary microcarcinoma under active surveillance [15]. In our study, as we are including patients with tumors larger than 1 cm in maximal diameter as well as microcarcinomas, a repeat ultrasound performed a few months later in patients whose tumors are suspected of growing, is considered prudent.

While multiple studies have demonstrated considerable intra-observer and interobserver variability of ultrasound measurement of thyroid nodules [16, 17], Ito et al. report that volume measurements are more prone to higher interobserver variation compared with maximal tumor diameter since two- or three-dimensional measurements are required for volume measurement [15]. Chung et al. reported interobserver difference of up to 24% in maximum diameter and 72% in PTC volume and suggested these values be taken into account to determine the cutoff for defining true growth of PTC during AS follow-up exams [18].

While studies have demonstrated that benign nodules may grow in size with time [19], others have exhibited that cancer nodules progress rapidly compared to benign nodules with growth of 2-3 mm/year [20, 21]. However, there is no evidence to suggest that growing PTCs on AS can lead to life threatening recurrences. Since prospective AS studies for PTCs have only been initiated in the recent past, to remain vigilant, experts have adopted a stringent cutoff of 3 mm increase or > 3 mm increase in size in any one dimension.

**Fig. 2:**
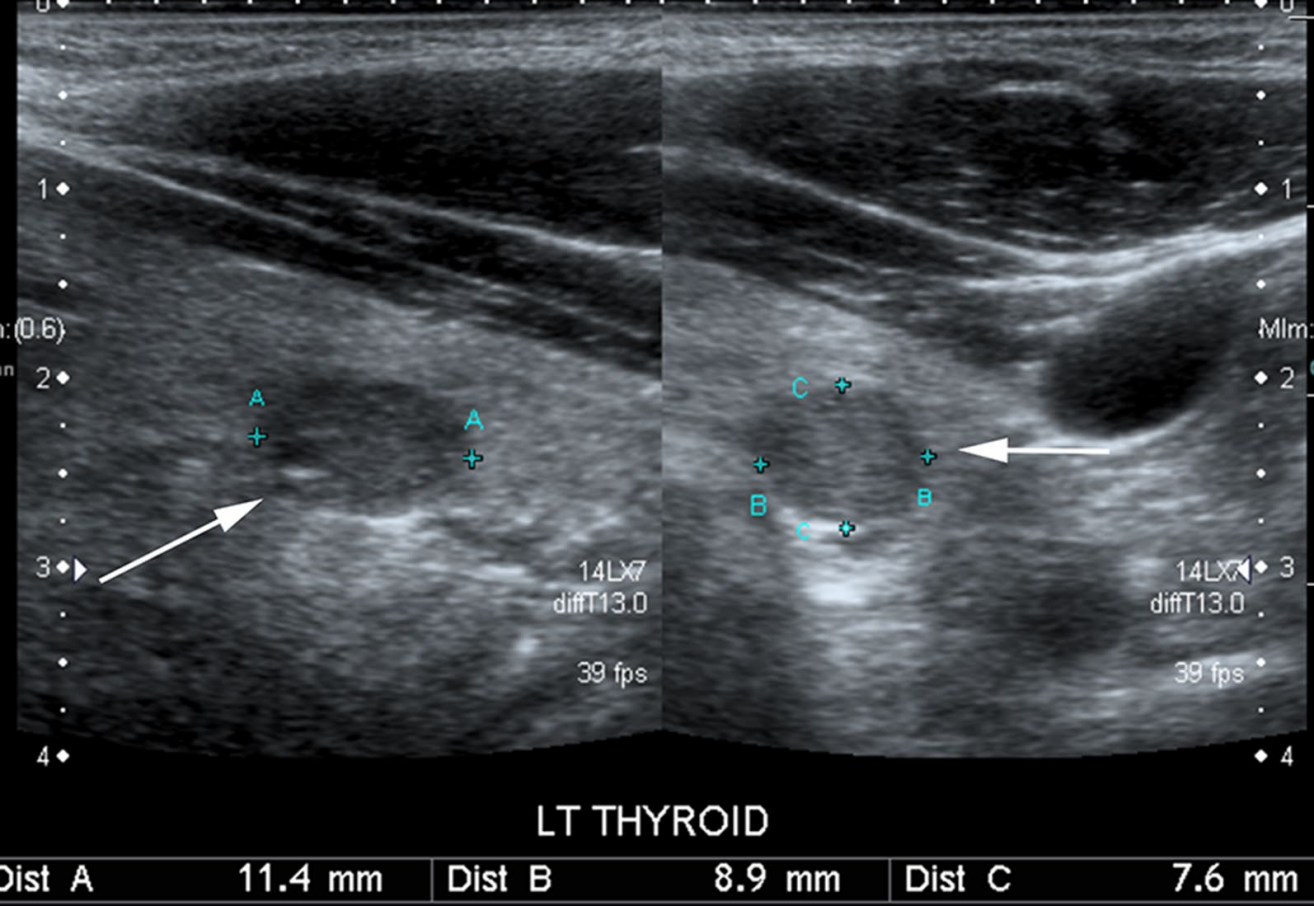
38-Year-old male with 11 mm PTC in left lobe of thyroid gland (arrow). The image depicts the consistent and reproducible three-axes technique to measure thyroid nodules described in ACR Thyroid Imaging, Reporting and Data System (TI-RADS). Measurements include the maximum dimension on an axial image (distance B on the image), maximum dimension perpendicular to the axial measurement on the same image (distance C) and the maximum longitudinal image on a sagittal image (distance A). Following the above protocol decreases the probability of incorrect measurements at AS follow-up scans performed by different operators in a busy clinical department of a large academic center

**Fig. 3:**
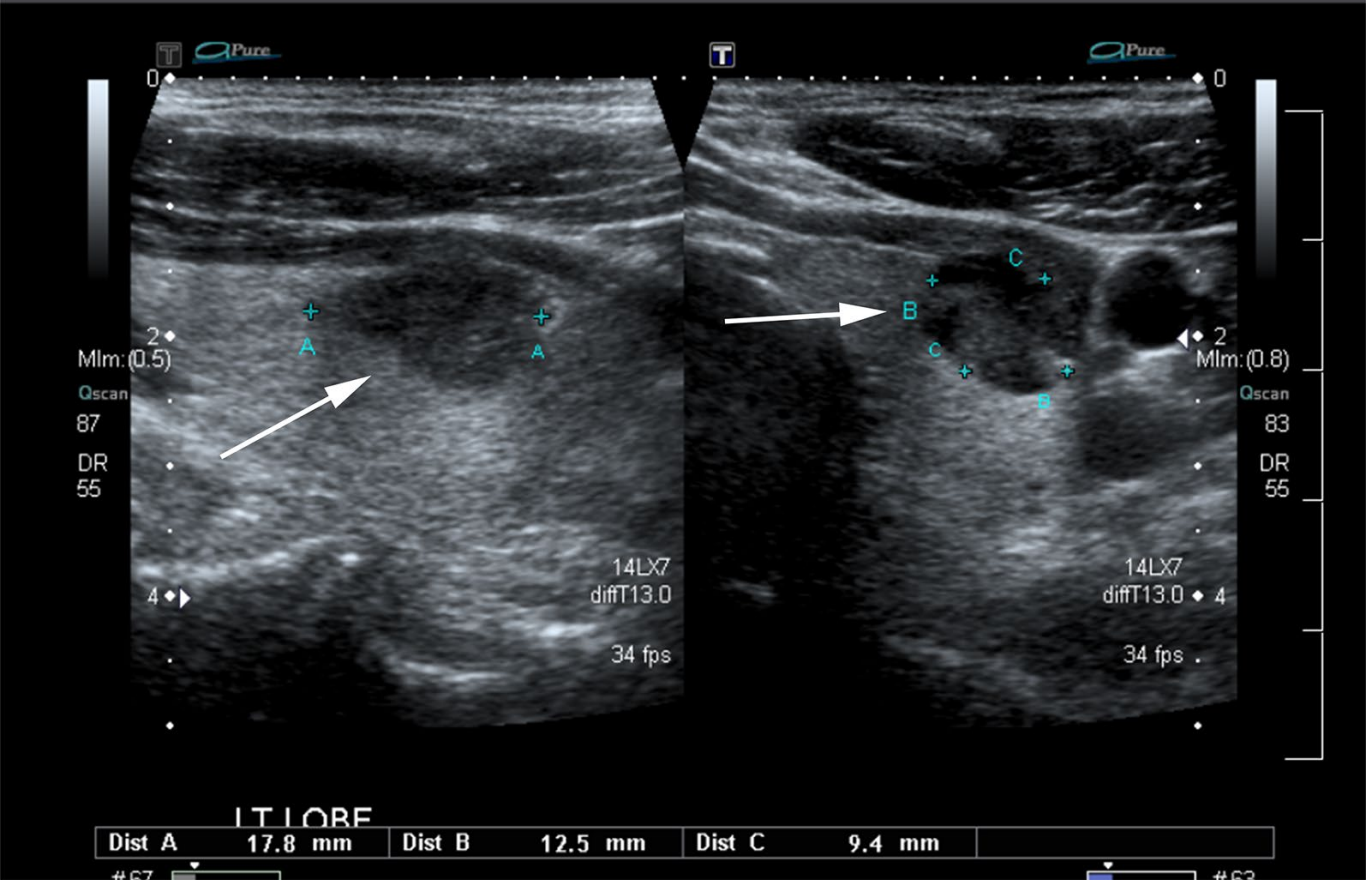
46-Year-old male with 18 mm obliquely oriented PTC in left lobe of thyroid gland (arrow). The image depicts the correct three-axes technique to measure thyroid nodules described in ACR TI-RADS. Distance B illustrates the maximum dimension of the PTC on the transverse image, while distance C represents the maximum dimension perpendicular to distance B plane on the image. Note that distance B and distance C on the image are measured in the axes of the nodule and not that of the thyroid gland. Distance A depicts the maximum longitudinal image on the sagittal image

**Fig. 4:**
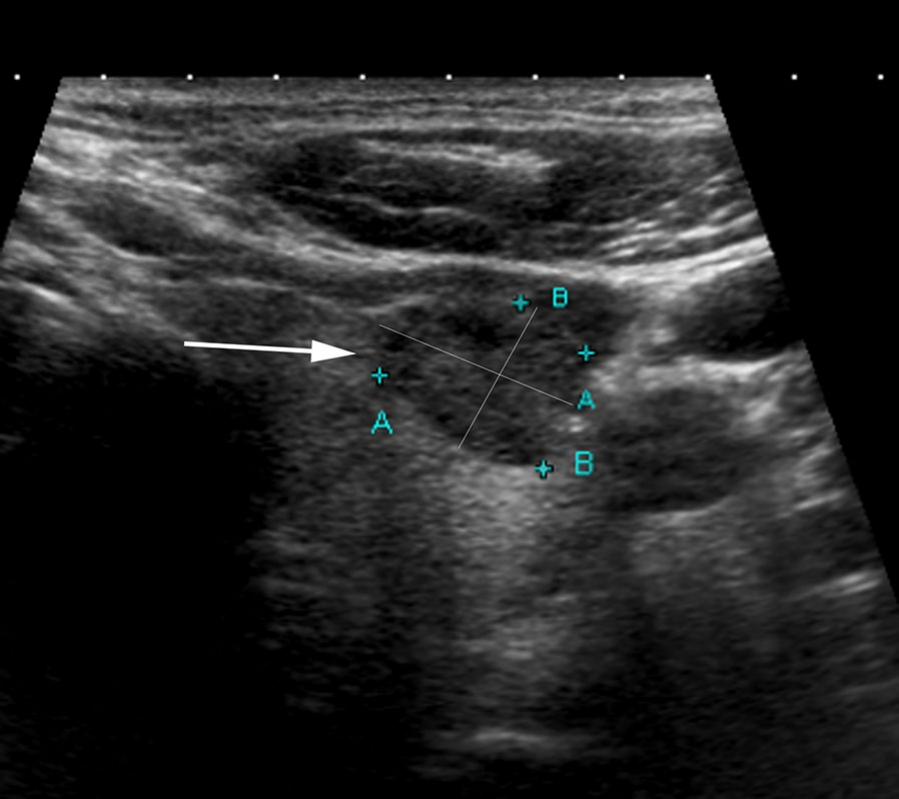
47-Year-old male with PTC in left thyroid lobe (arrow). The image illustrates incorrect placement of calipers for measurement of size (distances A and B on the image) of the PTC. Distance A and distance B have been measured in the plane of the thyroid gland, whereas ACR TI-RADS describes measurements should be recorded along the orientation of the nodule, i.e., axes of maximum dimension of the nodule in the transverse image and maximum dimension perpendicular to the first measurement. The two lines drawn perpendicular to one another in the image represent the accurate technique for measuring size of nodules. Inaccurate placement of calipers for measuring a PTC on AS at baseline or follow-up may lead to false impression of progression and discontinuation of AS

Accurate, consistent and reproducible measurements of PTCs are therefore essential in this group of patients. The ACR Thyroid Imaging, Reporting and Data System (TI-RADS): White Paper describes a three-axes technique to measure a thyroid nodule ensuring accurate and reproducible measurements [22]. Measurements include the maximum dimension on an axial image, maximum dimension perpendicular to the axial measurement on the same image and the maximum longitudinal image on a sagittal image (Fig. 2). The same measurement technique can be adopted for obliquely orientated nodules (Fig. 3). Following the above protocol decreases the probability of incorrect measurements at AS follow-up scans performed by different operators in a busy clinical department of a large academic center. Incorrect measurements could otherwise result in discrepant reports and in unnecessary surgical resection. Figure 4 demonstrates an example of incorrect caliper placement.

Apart from interobserver variability and accurate placement of calipers for reassessing size of nodule, there are other factors which may hinder accurate nodule size measurement at follow-up.

#### Background thyroid parenchyma

**Fig. 5:**
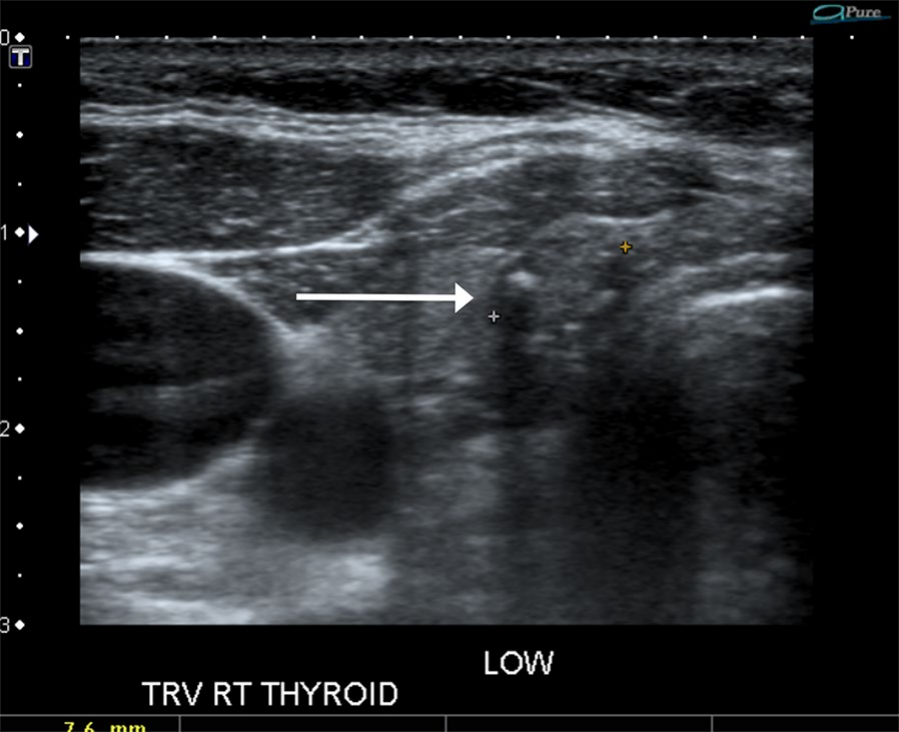
59-Year-old female patient with history of Hashimoto’s thyroiditis and small PTC in right thyroid lobe (arrow). The background thyroid parenchyma has a heterogenous echotexture which leads to loss of conspicuity of nodule margin and therefore limits accurate measurement of the nodule. PTCs in patients with thyroiditis and heterogenous background thyroid echogenicity should be assessed carefully to evaluate eligibility for AS

The echogenicity of the thyroid gland is normally higher than that of the adjacent strap muscles [23]. The majority of micropapillary carcinomas and PTCs < 2 cm are well-defined nodules and manifest as hypoechoic echogenicity compared to the background thyroid parenchyma, which allows precise caliper placement and accurate nodule size measurement at baseline and follow-up. However, in the setting of thyroiditis the background thyroid parenchyma is altered and may have a diffusely heterogenous echotexture. A decrease in thyroid echogenicity with inflammatory hypoechoic areas in the setting of Hashimoto’s thyroiditis (HT) or Grave’s disease can therefore lead to loss of conspicuity of nodule margins and limit accurate reproducible measurements of thyroid nodules (Fig. 5) [13]. This makes it even more critical in patients on AS where a size discrepancy of 3 mm in any one plane at follow-up can lead to a false interpretation of disease progression and discontinuation of AS. Therefore, PTCs in patients with thyroiditis and heterogenous background thyroid echogenicity should be assessed carefully to evaluate eligibility for AS.

#### Presence of calcification

**Fig. 6:**
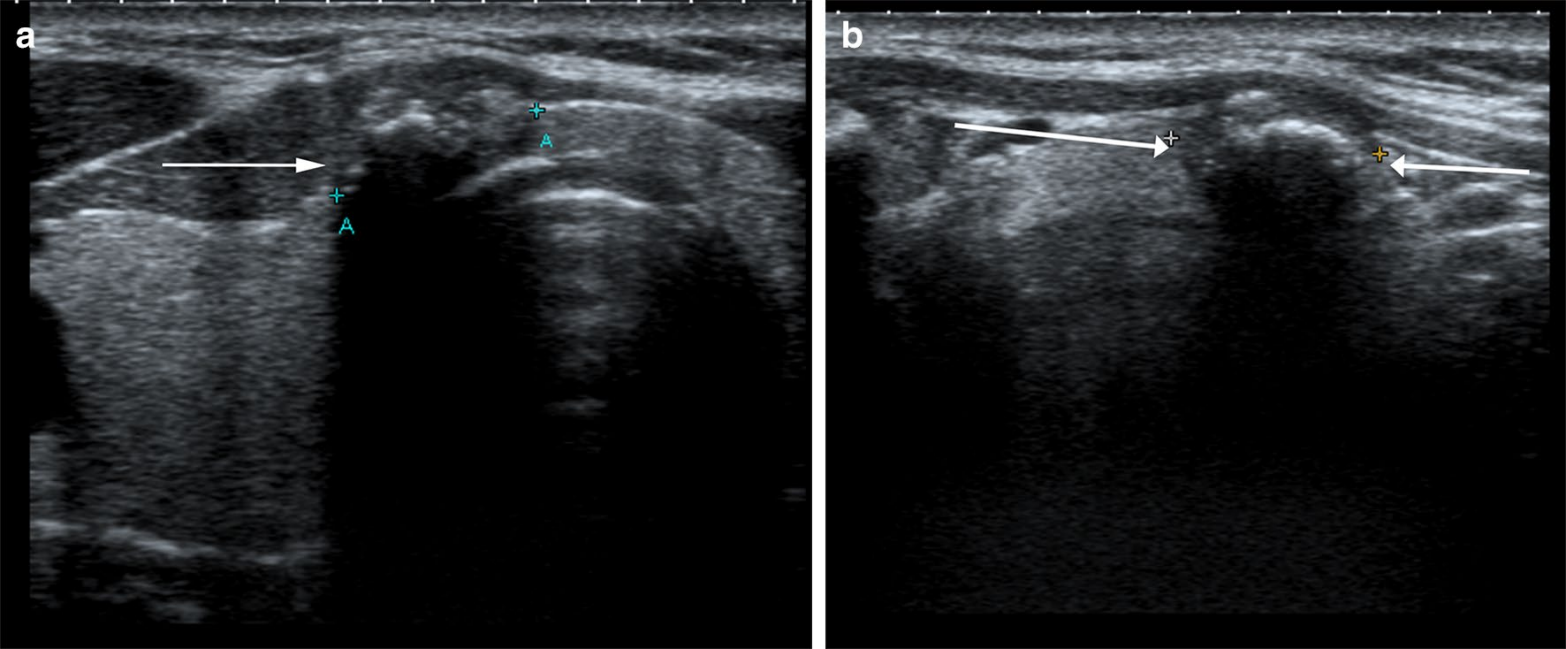
61-Year-old male patient. **a** (Transverse image) and **b** (sagittal image) show the PTC (arrow, within calipers) in right isthmus of thyroid gland. The nodule shows dystrophic coarse calcification with strong acoustic shadowing which limits assessment of posterior margin of the nodule. Not only does this preclude accurate measurement of the nodule at baseline or at follow-up, the angle of contact of nodule with trachea also cannot be assessed. In addition, the nodule shows extrathyroid extension anteriorly into strap muscles with loss of perithyroid echogenic line anteriorly. The patient was not offered AS and underwent surgery

Large irregularly shaped dystrophic or macro-calcification usually occurs secondary to tissue necrosis and may be present in up to a quarter of PTCs [24]. Coarse calcifications cause a strong acoustic shadowing that can limit assessment of the internal characteristics, including echogenicity and composition [22], and also the posterior margin of the nodule. In the setting of AS, this can preclude accurate placement of calipers and measurement of the nodule. While studies have reported that PTCs with dense calcification (coarse calcification or rim calcification) tend to not progress and are more likely to be indolent [2], the posterior acoustic shadowing can prevent accurate assessment of nodule margin posterior to the calcification to allow for nodule size comparison at follow-up. In our ongoing prospective observational study, we have excluded patients with known low-risk PTCs if dense calcification has obscured the posterior margin of nodule and prevented accurate measurement or assessment of tracheal and RLN involvement at baseline (Fig. 6). These patients are then referred for standard of care surgical excision of their known PTC.

### Presence of ETE

American Joint Committee on Cancer (AJCC) has defined minor and gross ETE separately [25]. Gross ETE is identified at the time of surgery and confirmed on histopathology, and is a negative prognostic indicator. It constitutes extension or involvement of strap muscles (T3b category) or invasion of subcutaneous soft tissues, larynx, trachea, esophagus or recurrent laryngeal nerve (RLN) (T4a category) with RLN and trachea involvement being most common. Minor or minimal ETE does not constitute a T3 category and is defined as ETE detected on histological examination [14]. For assessing ETE, ultrasound images should be evaluated for the following: (a) abutment, defined as lack of intervening thyroid tissue between the PTC and thyroid margin; (b) disruption, loss of perithyroid echogenic line at site of contact with the known cancerous nodule; (c) contour bulging; defined as outward bulging of thyroid contour by the nodule beyond expected margin; and (d) replacement of strap muscle, with indistinct margin with the strap muscle (for gross ETE).

**Fig. 7:**
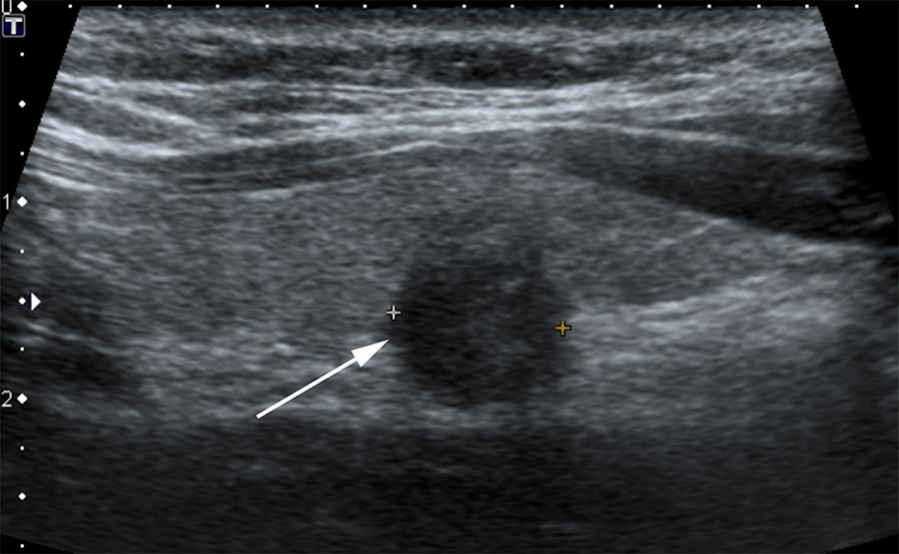
57-Year-old female patient with 8 mm PTC with extrathyroid extension (ETE). Sagittal image of the right lobe of thyroid gland shows the hypoechoic PTC extending beyond the margin of the gland posteriorly into the perithyroid connective tissue (arrow). The patient was not offered AS

**Fig. 8:**
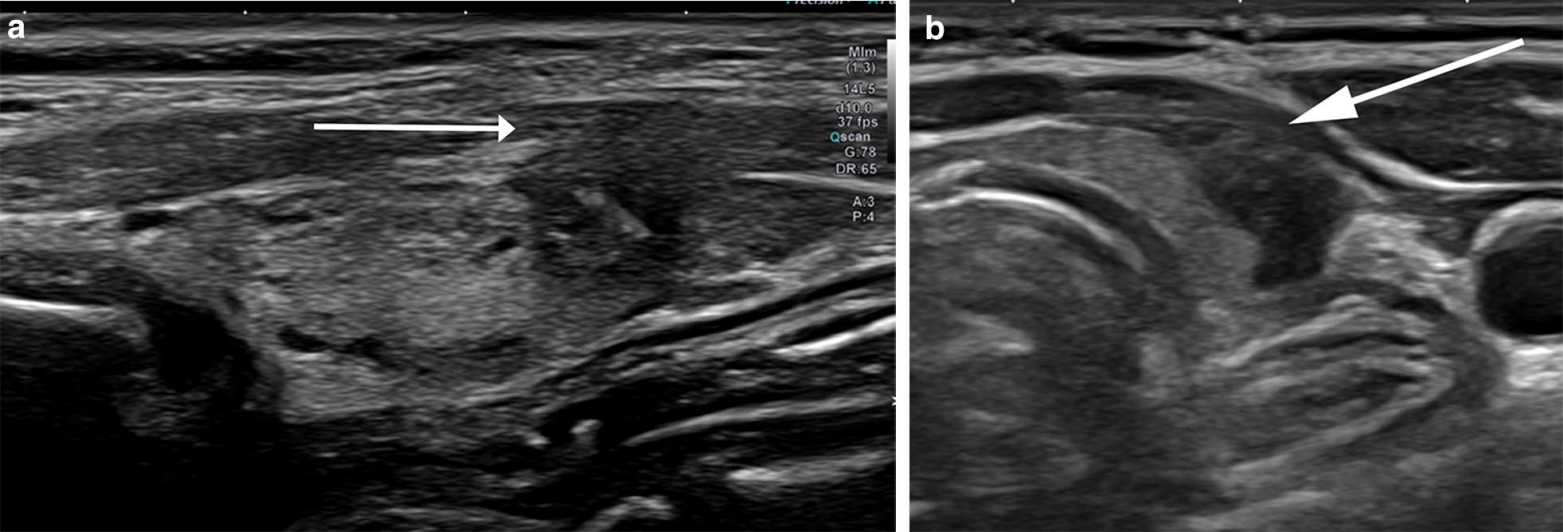
41-Year-old female patient. **a** (Sagittal image) and **b** (transverse image) shows a hypoechoic PTC in left thyroid gland with disruption and loss of perithyroid echogenic line anteriorly (arrow) at site of contact with the tumor suggesting minor ETE. The patient was not offered AS in our study and underwent surgery

While gross ETE to strap muscles is mostly visible on imaging (Fig. 7), minor ETE may often be difficult to ascertain on ultrasound. Of all the features above, capsular disruption with loss of perithyroid echogenic line at site of contact with the thyroid cancerous nodule (Fig. 8) has the highest diagnostic accuracy of predicting minor ETE [14]. In a study by Chung SR et al., only 27% (69/259) nodules with > 25% abutment of its perimeter with thyroid capsule without capsular disruption had minor ETE [14]. Even though minimal ETE is not considered to be an independent prognostic indicator for relapse-free survival [26, 27], these PTCs are not ideal for inclusion into AS protocols [14, 28]. Any nodule with ultrasound features of capsular disruption or definite sign of ETE is not included in our ongoing AS study. Additionally, we propose a multi-disciplinary approach for complex cases. PTCs with capsular abutment and/or bulge but without definite ETE or disruption of the perithyroid echogenic line are reviewed by the radiologist in conjunction with the study surgeon and endocrinologist. Factors such as patient compliance to follow-up scans, age, nodule size, in addition to the sonographic appearance of the nodule are taken into consideration to decide patient enrollment into study. This is particularly important in the research context wherein the patients are closely followed to detect any change in size or adverse sonographic features.

#### Relation of PTC to trachea and recurrent laryngeal nerve

The location of the PTC is one of the most important factors in determining if a patient is suitable for AS. The relationship of a PTC to the trachea and recurrent laryngeal nerve (RLN) is a determining factor for enrollment into AS. AS is contraindicated if imaging suggests that the PTC may be invading the trachea or the RLN. These tumors should be operated upon immediately.

**Fig. 9:**
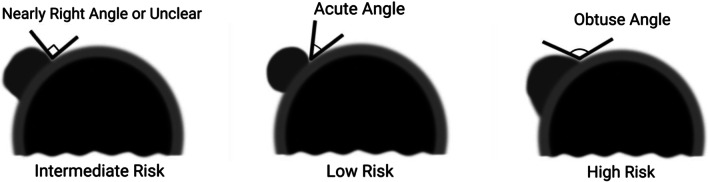
Diagram representing risk classification of tracheal invasion by PTC based on angle of contact between the tumor and the tracheal cartilage (Figure adapted from Miyauchi A. Clinical Trials of Active Surveillance of Papillary Microcarcinoma of the Thyroid. World J Surg. 2016 Mar;40(3):516–22. https://doi.org/10.1007/s00268-015-3392-y)

**Fig. 10:**
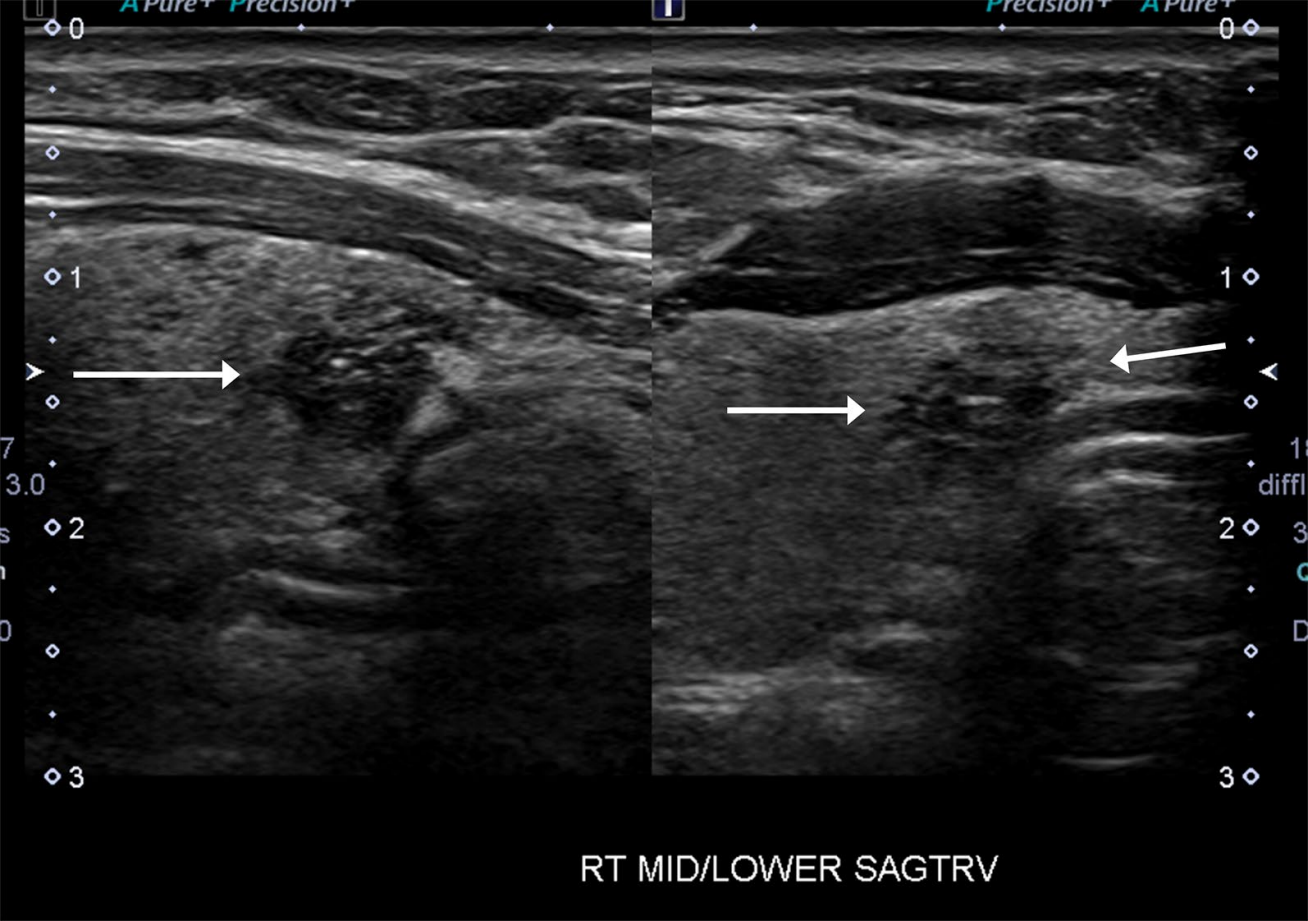
44-Year-old female with right lobe PTC. Sagittal and transverse images show that the hypoechoic PTC (arrows) has an acute angle of contact with the trachea. The patient was offered AS and continues to be monitored as per study protocol

**Fig. 11:**
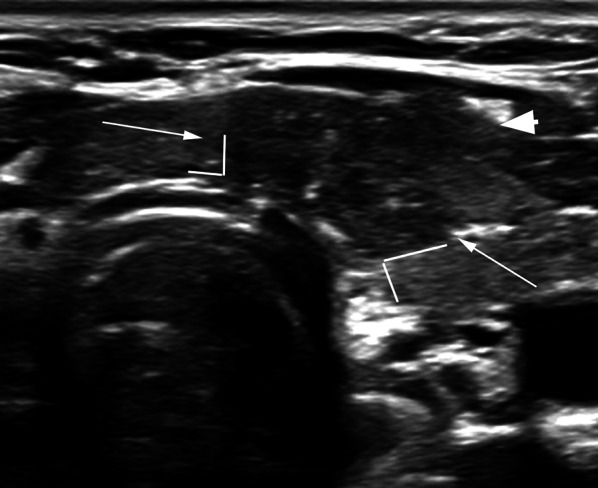
27-Year-old female with 14 mm PTC in left isthmus of thyroid gland. The nodule has a right angle/obtuse angle contact with the trachea which suggests a high risk of invasion into trachea. In addition, there is gross ETE noted anteriorly with disruption of the echogenic line and tumor extension to the strap muscles (arrowhead). The patient was not offered AS in our study and underwent surgery

Risk of tracheal invasion is assessed by the angle formed by the PTC and the tracheal cartilage (Fig. 9). Miyauchi et al. [29] described that the size of the PTC (≥ 7 mm) and its angle with the tracheal cartilage can predict tracheal invasion. In a series of patients who underwent surgery, none of the PTCs < 7 mm invaded the trachea, regardless of the angle of contact between the tumor and the trachea [30]. In cases of PTCs measuring ≥ 7 mm, the angle of contact between the PTC and the tracheal cartilage demonstrated a correlation with tracheal invasion on surgery. 24% of PTCs with obtuse angle between the nodule and the tracheal cartilage had invasion to trachea, while 17% with an unclear/right angle contact and 2% nodules with acute angle to the tracheal cartilage showed extension into peritracheal connective tissue but not to the tracheal cartilage [30]. Therefore, PTCs > 7 mm with an acute angle to the tracheal cartilage may be enrolled in AS (Fig. 10) [29]. While this study assessed PTCs < 1 cm, other studies with larger nodules have supported a similar finding [14], wherein the risk of ETE or invasion increased if the nodules formed an obtuse angle with the tracheal cartilage (Fig. 11). Chung et al. included 1656 thyroid cancers in their study (median size 1.3 cm) and reported that 12 of 18 nodules with an obtuse angle with the tracheal cartilage on ultrasound had tracheal invasion while none of the 265 nodules with acute angle and only 1 of 134 nodules with right angle contact with the tracheal cartilage had tracheal invasion at surgery [14].

**Fig. 12:**
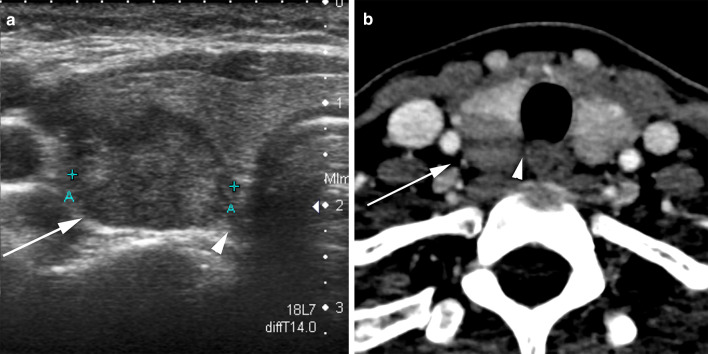
40-Year-old female with PTC in the posterior aspect of right lobe of thyroid gland. Transverse ultrasound image (**a**) shows a 13 mm PTC with loss of intervening normal thyroid parenchyma between the PTC and anatomic course of recurrent laryngeal nerve (RLN) (arrowhead). Corresponding axial contrast enhanced CT scan at the level of the PTC (**b**) again confirms the absence of intervening normal thyroid parenchyma between the PTC and the expected course of RLN (arrowhead) with protrusion of the nodule posteriorly beyond the margin of the gland. The patient was therefore not offered AS in our study

The relationship of the PTC to the RLN is an equally important consideration in evaluating eligibility for AS. Invasion of the RLN is associated with loss of intervening normal parenchyma between the PTC and anatomic course of the RLN [31] (Fig. 12a). Loss of normal thyroid tissue between the PTC and trachea-esophageal groove (TEG) with protrusion of the nodule into TEG or posteriorly is deemed as a high risk of RLN invasion. Ito et al. [30] also reported that PTC < 7 mm did not invade the RLN, regardless of the risk classification. Similarly, Chung et al. [14] reported that 20 of 58 nodules with protrusion into TEG required RLN resection or shaving at surgery. While US is the primary diagnostic test to assess relationship of the PTC to trachea and RLN, CT scan may occasionally be useful in assessment of nodules in posterior thyroid to evaluate relationship of the PTC to TEG and RLN [32, 33] (Fig. 12b).

### Assessment for nodes

Proper staging of PTC and inclusion into AS protocols relies on assessment of the neck and ruling out any suspicious cervical nodes. Microcalcification, cystic changes, rounded morphology, increased peripheral vascularity, spiculated margins and loss of fatty hilum are sonographic features suspicious for metastatic lymph nodes from thyroid cancer [34]. While some studies have reported minimum axial diameter of 6 mm as size cutoff for metastasis [35], any size cutoff may simply change sensitivity and specificity for detection of nodal metastases [36]. Microcalcification and/or cystic changes in cervical nodes are highly sensitive morphological features for malignancy in a patient with PTC and therefore should be considered as metastases until proven otherwise even if small in size [34].

### What the sonographer needs to know

The operator needs to be aware of the selection criteria and ultrasound appearances for case selection to AS [summarized in Table 1]. Also, meticulous US is mandatory at follow-up for patients on AS. Patients with PTCs within study inclusion size cutoff, confined to the thyroid gland without capsular disruption and located away from the trachea and RLN, are suitable for enrollment. PTCs that demonstrate nodule growth of > 3 mm in the largest dimension at any time point relative to the baseline measurement (confirmed on two consecutive ultrasounds) are presently discontinued from AS. If the PTC is < 7 mm and abuts the trachea or RLN they may stay in AS. If a PTC measuring ≥ 7 mm abuts the trachea, the angle of abutment should be interrogated. The angle of abutment is assessed by measuring the angle formed by the tumor margin and the tracheal cartilage. An acute angle of abutment is offered AS, and obtuse angle of abutment should be referred for surgery. Similarly, a PTC measuring ≥ 7 mm with a rim of thyroid between it and the RLN may remain in AS; if there is loss of the thyroid rim or protrusion of the nodule into TEG, the patient should be referred to surgery. Figures 5, 6, 7, 8, 11, 12 are examples of patients who did not meet AS criteria in our study and were referred for surgical excision.

**Table 1:** Key ultrasound features with clinical implications of the findings

Ultrasound feature	Clinical implication: active surveillance	Clinical implication: surgery
Nodule size	As per study inclusion criteria (< 2 cm in Clinicaltrials.gov: NCT03271892)	
Nodule measurement	Maximum dimension on an axial image, maximum dimension perpendicular to the axial measurement on the same image and the maximum longitudinal dimension on a sagittal image	
Nodule location	Ensure that PTC is identified and labeled accurately for follow-up, especially in patients with MNG and multiple similar sized nodules	
Evaluation for extrathyroidal extension (ETE)	Confined to the thyroid parenchyma	Loss of perithyroid echogenic line at site of contact of PTC
	Thyroid margin bulge without disruption of perithyroid echogenic line	
Nodule relationship to the trachea	< 7 mm nodule irrespective of relationship to trachea	–
	≥ 7 mm nodule if acute angle to the trachea	≥ 7 mm if obtuse angle to the trachea
Nodule relationship to tracheo-esophageal groove (TEG)	< 7 mm nodule irrespective of relationship to TEG	–
	≥ 7 mm nodule if thyroid rim present between the PTC and TEG	Lacking normal thyroid rim between TEG and PTC or protrusion of nodule to TEG or posteriorly
Evaluation of lymph nodes	No suspicious cervical lymph nodes	Lymph node(s) suspicious for metastatic disease (require biopsy confirmation)
Nodule growth at follow-up	< 3 mm in any one plane	≥ 3 mm in any one plane or maximal diameter
		(> 3 mm in Clinicaltrials.gov: NCT03271892)

### Other relevant ultrasound features

Nodule calcification, vascularity and margin all have diagnostic relevance for patients enrolled in AS studies. Nodules with strong calcification typically in older patients have been shown to have a lower incidence of tumor enlargement compared to nodules with weak or absent calcification [8]. Fukuoka et al. reported nodules that develop rim calcification generally do not progress [2]. Interestingly, Fukuoka et al. also reported that nodules demonstrating strong vascularity at the beginning of AS had a higher rate of growth but that vascularity diminishes on follow-up ultrasounds [2]. Younger patients have a higher risk of progression compared to the older cohort, and the rate of nodule calcification correlates with patient age. Ill-defined nodule margins are described as an aggressive feature of PTC nodules. Ito et al. reported a higher incidence of tumor recurrence with higher rates of lateral node metastasis in patients with ill-defined nodule margins compared to well-defined nodules. However, since no life threatening recurrences were noted in the study by Ito et al. [37], PTCs with ill-defined margins are not excluded from enrollment into AS studies at this time. These ultrasound features, however, should be documented during AS and follow-up scans should meticulously look for any suspicious lateral neck nodes.

## Conclusion

Meticulous ultrasound is paramount to AS clinical research in PTC. As many institutions initiate AS studies to assess whether low-risk PTCs can safely be followed rather than subjected immediately to surgery, constraints around ultrasound evaluation of PTCs can limit appropriate enrollment and accurate follow-up. In this review, we highlight the PTC and background thyroid characteristics which may limit adequate assessment and outline technique for accurate ultrasound evaluation for AS eligibility and follow-up. It is anticipated that future advances in ultrasound technology, data storage and use of modalities such as artificial intelligence will enable progress in effective implementation of AS in a broader range of clinical practice settings.

## Data Availability

The datasets used and/or analyzed during the current study are available from the corresponding author on reasonable request.

## Abbreviations

ACR TI-RADS: American College of Radiology—Thyroid Imaging Reporting and Data System
AS: Active surveillance
ETE: Extrathyroid extension
PTC: Papillary thyroid cancer
RLN: Recurrent laryngeal nerve
US: Ultrasound
